# Outcomes of surgical hip dislocation combined with bone graft for adolescents and younger adults with osteonecrosis of the femoral head: a case series and literature review

**DOI:** 10.1186/s12891-022-05456-w

**Published:** 2022-05-26

**Authors:** Wenhuan Chen, Jianxiong Li, Wenxuan Guo, Shihua Gao, Qiushi Wei, Ziqi Li, Wei He

**Affiliations:** 1 The Third Clinical Medical School, Guangzhou University of Chinese Medicine, Jichang Road 12#, District Baiyun, Guangzhou, Guangdong China; 2 The First Clinical Medical School, Guangzhou University of Chinese Medicine, Jichang Road 12#, District Baiyun, Guangzhou, Guangdong China; 3 The First Clinical College, Zhejiang Chinese Medical University, Hangzhou, Zhejiang China; 4 Department of Joint Diseases, Traumatology & Orthopedics Institute of Guangzhou University of Chinese Medicine, The Third Afliated Hospital of Guangzhou University of Chinese Medicine, Guangzhou, Guangdong China

**Keywords:** Osteonecrosis of the femoral head, Adolescent and younger adults, Surgical hip dislocation, Hip preservation, Case series

## Abstract

**Background:**

Osteonecrosis of the femoral head (ONFH) may occur in the adolescent and younger adults (AYAs). Total hip arthroplasty (THA) is not the best treatment option for younger patients. Surgical hip dislocation (SHD) combined with bone graft can be used in patients at different stages to reconstruct the bone structure in the head and delay the replacement time. The purpose of this study was to evaluate the effect and potential influencing factors of this surgery for ONFH in AYA patients.

**Methods:**

We conducted a literature review and a retrospective research of our own cases. The Pubmed, Cochrane Library, EMBASE and CNKI databases were searched from 1 January 2001 to 1 October 2021, for clinical studies. A retrospective case series study of 34 patients (38 hips) treated with SHD combined with bone graft was performed.

**Results:**

A total of 13 studies were included and the results showed that SHD combined with bone grafts had better clinical results for patients with pre- or early post-collapse. In the case series study, we retrospectively analyzed 34 patients (38 hips), and the mean follow-up time was 40.77 ± 15.87 months. One patient died and three patients were converted to THA finally. The post-collapse degree and post-lesion size were better than those before the operation (*P* < 0.05). The iHOT-12 at the last follow-up was significantly higher than that before the operation (*P* < 0.05). There were significant differences in the results of hip Harris score (HHS), visual analogue scale (VAS) and Western Ontario and McMaster Universities Osteoarthritis Index (WOMAC) before the operation, 2 years after the operation and at the last follow-up, but the difference was not related to the follow-up time (*P* < 0.05). There were no significant differences in the final clinical score and arthritic changes among different Japanese Investigation Committee (JIC) classification, the degree of collapse and the size of the necrotic (*P* > 0.05).

**Conclusions:**

In AYA patients, SHD combined with bone grafting is a potentially good option for hip preservation in ONFH. The differences in JIC classification, collapse degree and lesion size did not affect the final clinical function and the risk of osteoarthritis. Even for very severe cases at collapsed stage, good short-term clinical effects can still be achieved by SHD combined with bone graft.

**Trial registration:**

ChiCTR2100055079.retrospectively registered.

**Supplementary Information:**

The online version contains supplementary material available at 10.1186/s12891-022-05456-w.

## Background

Osteonecrosis of the femoral head (ONFH) is a potentially disabling disease [1]. With the development of society and the improvement of diagnosis and treatment technology, the incidence of ONFH is higher, and patients are younger. After using steroids to treat underlying diseases, one-third of children develop ONFH [2–9]. If interventions cannot be taken in time, 80% of patients will have femoral head collapse, which will eventually lead to total hip arthroplasty (THA) [10]. It has been reported in literature that approximately 75% of hip replacements can last 15 to 20 years, and only 50% can last 25 years [11], while in adolescent and younger adults (AYAs) [1], the longest follow-up time after hip arthroplasty is less than ten years on average. Therefore, it is more valuable for young patients to choose appropriate hip-preserving surgery to delay or even avoid THA.

The surgical hip dislocation (SHD) approach was first described by Professor Ganz et al. [12] as a safe approach for the treatment of internal hip joint injuries, which can fully expose the femoral head and rarely cause complications related to the blood supply of the femoral head [12, 13]. Compared with the traditional surgical approach, it preserves the medial circumflex femoral artery, reduces the damage to the blood supply of the femoral head, and at the same time fully exposes the femoral head through dislocation, which is conducive to intraoperative procedures. More conducive to reduction and fixation of fracture fragments in femoral head fractures [14] An increasing number of doctors have combined this approach with the traditional hip-preserving operation, that is, decompression and impacting bone graft, which has been applied to adult ONFH in the early or middle stage and achieved good results [15–19]. Seyler et al. [20] recommended performing hip preservation surgery with nonvascularized bone grafts in precollapse or early collapsed patients with less than 2 mm of head depression or a small lesion. However, for AYA patients with severe ONFH, it remains unclear whether this type of hip preservation surgery is appropriate.

The purpose of this study was to observe the clinical effect of SHD combined with bone grafts for ONFH in AYA patients. The primary outcome was postoperative iHOT-12 score. The secondary aim was to analyse the factors that may influence the results. We assume that AYA patients with severe collapse or extensive lesions can also obtain good clinical outcomes through this operation.

## Methods

### Review of literature: search strategy

We conducted a literature review of research regarding hip preservation treatment of patients with ONFH using the SHD approach. Two researchers independently searched PubMed, EMBASE, Cochrane Library and China National Knowledge Infrastructure (CNKI) from January 1, 2001 to October 1, 2021, using the keywords “surgical hip dislocation”, “osteonecrosis of the femoral head”, and “hip preservation”. The inclusion criteria were as follows: (1) patients were diagnosed with ONFH; (2) the treatment method was hip preservation surgery; (3) the surgical hip dislocation approach was adopted; (4) language was English or Chinese; and (5) outcome indicators were the clinical score for evaluating hip joint function and the classification of the disease. The exclusion criteria were as follows: (1) review articles; (2) case reports; (3) incomplete or unavailable data results; and (4) patients who underwent surgery again.

The following data were extracted: patient characteristics (age, pathogenic factors), clinical manifestations (preoperative classification, postoperative hip Harris score) and treatment (operation, complications and THA rate). The two researchers independently evaluated the retrieved articles by reading the title and abstract and evaluated all the articles that might meet the requirements by obtaining the full text. Any differences between the two researchers were settled through discussion.

### Case series

#### Inclusion and exclusion criteria

This study was a descriptive case series without a control group. A retrospective study was conducted based on the hospital's clinical medical records and radiological data with the approval of the ethics committee. The diagnosis and evaluation were based on history, physical examination, radiological data, and limb function score. The inclusion criteria were as follows: (1) 12 ~ 30 years; (2) diagnosis of ONFH; (3) no cerebrovascular or nervous system diseases; and (4) treatment with SHD combined with bone graft. The exclusion criteria were as follows: (1) those who had previously undergone any hip preservation surgery; (2) those who needed to continue taking steroid hormones because of other diseases; and (3) incomplete or missing follow-up data.

#### Therapeutic method

All operations were performed by the same surgeon, and all patients were treated with SHD combined with iliac bone grafts. The Ganz approach (modified K-L approach) was adopted [21]. After completing the osteotomy of the greater trochanter, the attached muscles such as the gluteus minimus were retained, and the bone fragment was pulled anteriorly and superiorly to expose the joint capsule. The joint capsule was cut in an arc and it aims to protect the deep branch of the medial circumflex femoral artery and the acetabular labrum. The femoral head was dislocated from the front through hip flexion. After dislocation of the femoral head, a 1 × 1.5 cm bone window was formed at the head-neck junction. A strip of cortical cancellous bone was grafted from the iliac crest. According to the size of the lesion, various drill bits of different diameters combined with high-speed burr was used to remove the necrotic bone. Then, the autologous cancellous bone was tightly pressed. The collapsed articular surface was restored the sphericity of the femoral head, and then the free iliac bone flap is trimmed and embedded into the bone window to support the autologous cancellous bone graft and the articular surface of the femoral head. If necessary, the iliac bone was fixed with screws to prevent loosening [17]. All patients received rehabilitation and physical therapy after surgery. In the first six months after the operation, bilateral axillary crutches were used to keep the affected limbs from weight bearing. For the next six months, patients used a crutch to partially load the affected limb. Then, when approved by the doctor’s assessment, the patient could use the affected limb to bear the full weight. Since the complications of AYA patients were unclear, we did not use any anti-osteoporosis drugs and used nonsteroidal anti-inflammatory drugs or analgesics for symptomatic treatment.

#### Follow-up and assessment

Radiological follow-up was performed at 1, 3, 6, and 12 months postoperatively, and outpatient review was conducted once a year. Postoperative complications were recorded, including superficial wound infection, heterotopic ossification, nerve injury, lower extremity venous thrombosis, internal fixation loosening, delayed fracture union or nonunion, etc. According to the radiological data of the patients before the operation and at the last follow-up, the collapse degree and lesion size were analysed. The index of collapse degree refers to the 2019 Revised Version of Association Research Circulation Osseous Staging System of Osteonecrosis of the Femoral Head [22]. The doctor recorded the results of hip Harris score (HHS), visual analogue scale (VAS), Western Ontario and McMaster Universities Osteoarthritis Index (WOMAC), and iHOT-12 before the operation, 2 years after the operation, and at the last follow-up, and evaluated the osteoarthritis of the patient's hip joint based on the radiographic film at last follow-up.

The degree of osteoarthritis was evaluated according to the Tonnis grade. Grade 0 indicates no degenerative change; Grade 1 indicates mild joint space stenosis, mild sclerosis and mild marginal osteophyte; Grade 2 indicates moderate joint space stenosis, moderate sclerosis of femur or acetabulum, and small subchondral cyst of femur or acetabulum; Grade 3 indicates that the joint space is obviously narrow (< 1 mm) or the joint space is reduced, and there is a large subchondral cyst on the femur or acetabulum.

#### Statistical analysis

The Mann–Whitney rank sum test was used to compare the overall distribution of lesion size and collapse degree before and after the operation. The differences in the HHS, VAS and WOMAC before the operation, 2 years after the operation and at the last follow-up were compared by one-way repeated-measures ANOVA. A paired t test was used to compare the preoperative and postoperative differences in the iHOT-12 score. The distribution of Tonnis classification at the last follow-up is reflected by a pie chart. Subgroup analysis was performed according to the Japanese Investigation Committee(JIC) classification, collapse degree and lesion size. And the VAS, Harris, WOMAC, iHOT-12 and Tonnis grade at last follow-up between different subgroups were compared using independent samples t-test, Kruskal–Wallis H test, one-way ANOVA and Fisher's exact test. The variables with *P* < 0.05 indicated statistical significance. The statistical analysis was performed using SPSS software v25.0 (SPSS Inc, Chicago, Illinois, U.S.A).

## Results

### Review of the literature

A total of 1091 related studies were obtained in the preliminary examination. The two researchers included 95 articles by reading the titles and abstracts. After reading the full text and screening according to the inclusion and exclusion criteria, 13 studies [15–19, 23–30] were finally included, and a total of 338 hips treated by SHD were extracted. Detailed information on literature is shown in Table 1. PRISMA flow diagram and checklist are shown in supplementary materials 1. We arranged and descriptively analyzed the retrieved literature data. In the 13 studies, only five had follow-up for more than two years. THA rates were reported in 8 studies, 3 of which reported THA cases within 2 years after surgery, and the overall THA rates reported in literature were 0–17%. Five studies reported postoperative complications, including delayed healing of the greater trochanter, nerve injury, heterotopic ossification, and superficial wound infection. The average age of patients in the included studies was approximately 30 years, only two studies included patients under the age of 18, and all patients were pre-collapse or early post-collapse. In terms of clinical function scores, that was Hip Harris score, most studies reported good results, only the score reported by Du et al. was lower than 80. However, there have been no reports on its clinical effects in younger patients with severe necrosis.

**Table 1:** Detailed summary of the literature review

Author	Patients	Mean age(year)	Interventions	ARCO classification	Pathogenesis(hips)	Follow-up time(m)	Post-HHS	THA	Complications
Shen et al. 2021 [23]	53	31.7(13–52)	SHD + Iliac bone grafts	II, III	Steroid 24 Alcohol 14 Trauma 9 Idiopathic 6	20(8–38)	83.2 ± 5.8	2	0
Liang et al. 2021 [24]	28	36.25(21–53)	SHD + Iliac bone grafts	II, III	Steroid 12 Alcohol 10 Idiopathic 6	24.29	87.07 ± 1.69	3	1
Tang et al. 2020 [25]	26	36.2(21–53)	SHD + PRP	II, III	Steroid 15 Alcohol 8 Idiopathic 3	36.6(26–42)	87.38 ± 8.21	1	1
Steppacher et al. 2020 [26]	13	29(20–49)	SHD	II, III	Steroid 4 Trauma 1 Idiopathic 8	36(12–84)	None^a^	1	2
Wei et al. 2020 [17]	32	27.6(18–49)	SHD + Iliac bone grafts	III	Steroid 18 Alcohol 5 Trauma 4 Idiopathic 5	41.0(29–51)	82.1 ± 4.6	1	0
Xia et al. 2020 [18]	33	31.7(19–46)	SHD	III	Non-traumatic	18(12–28)	81.50 ± 8.81	1	0
Zheng et al. 2020 [27]	15	32.3(23–50)	SHD + Iliac bone grafts	III	None	15.3(9 ~ 30)	83.75 ± 7.62	0	0
Du et al. 2019 [28]	15	32.76(17–46)	SHD + TCM	III	None	14.60(6 ~ 25)	74.37 ± 6.46	0	0
Sun et al. 2019 [16]	28	35.7(18–45)	SHD	III	Steroid 15 Alcohol 9 Idiopathic 4	25.8(12–48)	88.74 ± 1.68	5	1
Deng et al. 2018 [29]	43	36.8	SHD	II	None	21.8(14–36)	89.89 ± 8.13	0	0
Liu et al. 2018 [30]	22	34.3	SHD + PRP	II, III	Non-traumatic	12.40	86.84 ± 6.44	0	0
Zhuang et al. 2017 [19]	22	38.8(29–46)	SHD + TCM	III	Steroid 4 Alcohol 16 Trauma 2	11.8(6–18)	91.0 ± 6.3	0	0
Yao et al. 2017 [15]	8	36(26–44)	SHD	III	Steroid 3 Alcohol 4 Trauma 1	18.6(5–29)	80.53 ± 7.62	1	1
Chen et al. 2022	34	22.18(12–30)	SHD + Iliac bone grafts	III, IV	Steroid 10 Alcohol 16 Trauma 9 Idiopathic 3	40.77(22.33–74.07)	86.76 ± 10.22	3	0

*PRP* Platelet-Rich Plasma, *SHD* Surgical Hip Dislocation, *TCM* Traditional Chinese Medicine, *HHS* Hip Harris Score, *THA* Total Hip Arthroplasty, *ARCO* Association Research Circulation Osseous;

^a^This paper uses Merle d’Aubigné-Postel score to evaluate the pain and function

### Results of case series

In this continuous case series, a total of 117 patients with ONFH were treated with surgery at the First and Third Affiliated Hospital of Guangzhou University of Chinese Medicine between March 2015 and June 2019. Finally, 34 patients (38 hips) were included in the study according to the criteria. There were 22 males and 12 females with a mean age of 22.18 ± 5.89 (12–30) years old and a mean body mass index (BMI) of 20.44 ± 3.19 (kg/m^2^). The average follow-up time was 40.77 ± 15.87 (22.33–74.07) months. The patient's aetiology included trauma, steroids, alcohol, and idiopathic factors. The number of patients before surgery according to the JIC classification, collapse degree and lesion size are listed in Table 2. One patient died because of underlying disease, and three patients were converted to THA due to exacerbation. The details of 3 patients are shown in Table 3. The mean operation time was 128.16 ± 33.195 (100–180) minutes for each limb, and the mean intraoperative blood loss was 287.11 ± 99.374 (200–500) ml (Table 2). Complications had not been observed in any patients.

**Table 2:** Patient demographic characteristics. BMI, Body mass index; JIC, Japanese Investigation Committee

Demographics	Values
Gender (male:female)	22:12
Age (year)	22.18 ± 5.89(12–30)
BMI (kg/m.^2^)	20.44 ± 3.19
JIC classification (hips)	
Type C1	7
Type C2	31
Operation time (min)	128.16 ± 33.195(100–180)
Blood (ml)	287.11 ± 99.374(200–500)
Follow-up time (month)	40.77 ± 15.87(22.33–74.07)
THA	3
Death	1
Etiology(hips)	
Trauma	10
Steroid	16
Alcohol	9
Idiopathic	3
Collapse degree (hips)	
≤ 2 mm	16
2-4 mm	14
> 4 mm	8
Lesion size (hips)	
< 15%	3
15–30%	24
> 30%	11

**Table 3:** Details of three patients who converted to THA

Patient	Gender	Age(years)	Etiology	Hip	JIC	Collapse degree(mm)	Lesion size%	Pre-VAS	Pre-Harris	Pre-Womac	Pre-iHOT-12	Time toTHA(m)
NO.1	male	25	Alcohol	right	C2	2–4	> 30	8	76	14	52	11
NO.2	male	27	Alcohol + Steroid	right	C2	2–4	> 30	6	65	15	43	20
NO.3	male	30	Alcohol	left	C2	2–4	15–30	6	70	20	47	12

**Fig. 1:**
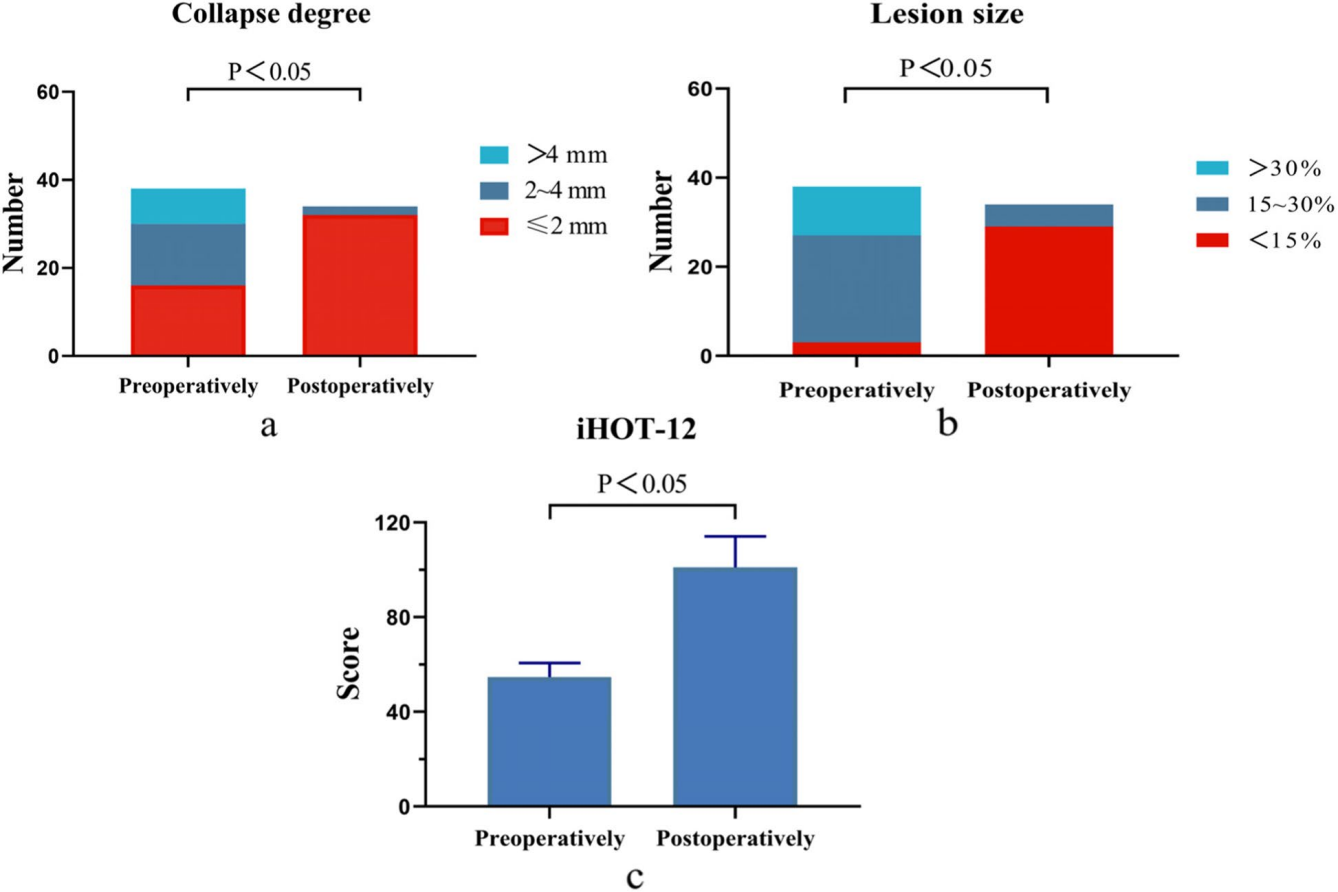
The overall distribution of preoperative and postoperative clinical outcomes of patients. **a** The results of collapse degree of femoral head. **b** The results of lesion size about femoral head necrosis area. **c** The results of iHOT-12 scores

Postoperatively, the collapse degree of 32 hips was ≤ 2 mm, while that of 2 hips were 2 to 4 mm. The lesion size of 29 hips was < 15%, and that of the other 5 hips were 15%—30%. The overall distribution was significantly better than that before the operation (*P* < 0.05). At the last follow-up, the iHOT-12 score was 101.09 ± 13.05, which was significantly higher than the preoperative score (54.71 ± 5.93, *P* < 0.05) Fig. 1.

**Fig. 2:**
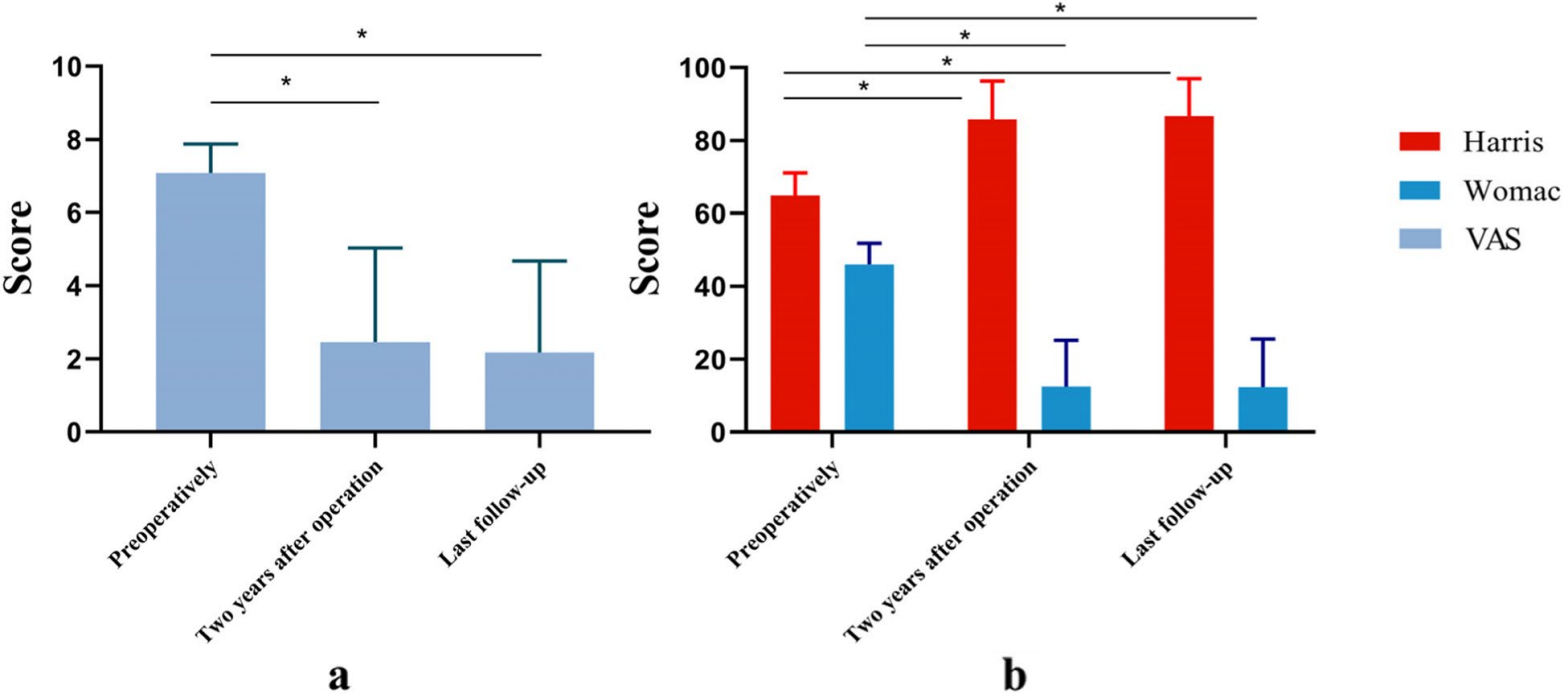
The results of HHS, VAS and WOMAC before operation, 2 years after operation and at the last follow-up.*indicates *P* < 0.05

The HHS, VAS and WOMAC scores before the operation were 64.85 ± 6.31, 7.09 ± 0.79 and 46.00 ± 5.91, the results at 2 years after the operation were 85.85 ± 1041, 2.47 ± 2.57 and 12.56 ± 12.78, and the results at the last follow-up were 86.76 ± 10.22, 2.18 ± 2.5 and 12.50 ± 13.24, respectively. The pre-HHS, pre-VAS and pre-WOMAC scores were significantly different from those at 2 years after the operation and at the last follow-up (*P* < 0.05). However, there were no significant differences between 2 years after the operation and the last follow-up (*P* > 0.05) Fig. 2.

**Fig. 3:**
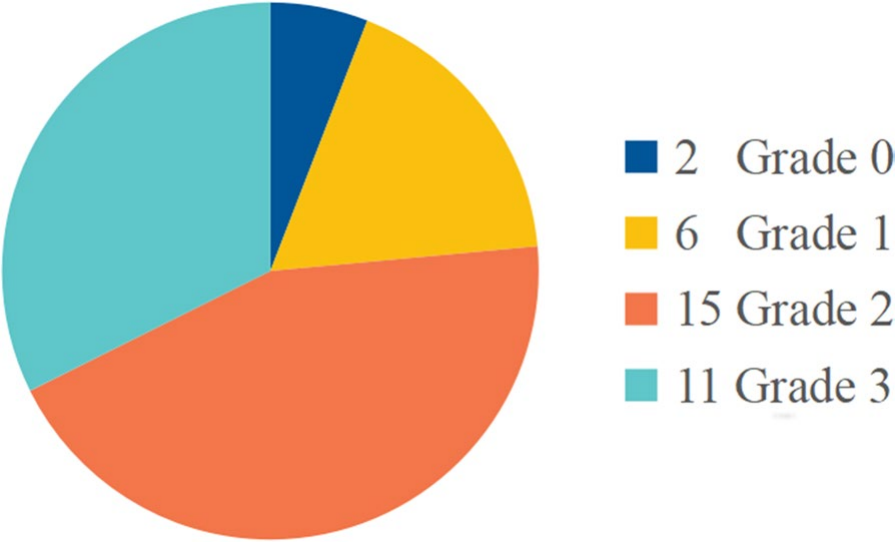
Distribution of Tonnis grade at the last follow-up (total = 34)

At the last follow-up, the images of the patients were evaluated, and the Tonnis grade was obtained through anteroposterior X-ray films. There were two hips with osteoarthritis grade of 0, six hips with a grade of 1, 15 hips with a grade of 2, and 11 hips with a grade of 3 (Fig. 3).

The results of the subgroup analysis are shown in Table 4.

**Table 4:** Results of Subgroup Analysis according to the different JIC classification, collapse degree and lesion size

	**JIC classification**		**Collapse degree**			**Lesion size**		
	C1	C2	≤ 2 mm	2-4 mm	> 4 mm	< 15%	15–30%	> 30%
VAS	2.29 ± 2.87	2.15 ± 2.46	3.06 ± 3.02	1.64 ± 1.86	1.00 ± 1.29	0.67 ± 1.16	2.45 ± 2.56	2.00 ± 2.69
Harris	82.71 ± 10.75	87.81 ± 10.02	82.81 ± 11.77	89.18 ± 7.73	92.00 ± 6.68	89.00 ± 10.15	85.68 ± 11.32	88.67 ± 7.75
WOMAC	18.29 ± 12.93	11.00 ± 13.14	19.19 ± 15.83*	7.91 ± 6.73	4.43 ± 5.35*	10.67 ± 11.59	13.18 ± 14.29	11.44 ± 12.24
iHOT-12	98.14 ± 14.31	101.89 ± 12.90	95.81 ± 14.77	106.45 ± 10.41	104.71 ± 8.30	109.33 ± 12.50	99.41 ± 13.71	102.44 ± 11.61
Tonnis(hips)								
Grade 0	1	1	2	0	0	1	0	1
Grade 1	0	6	2	2	2	0	5	1
Grade 2	3	12	5	7	3	0	11	4
Grade 3	3	8	7	2	2	2	6	3
P^a^	0.364		0.542			0.152		

^a^means taking Fisher’s exact test

^*^P < 0.05

There were no significant differences in VAS results at the last follow-up between C1 group (2.29 ± 2.87) and C2 group (2.15 ± 2.46, *P* > 0.05). The VAS results at the last follow-up were 3.06 ± 3.02, 1.64 ± 1.86 and 1.00 ± 1.29 for the collapse degree ≤ 2 mm, 2–4 mm and > 4 mm, respectively, and there were no significant differences (*P* > 0.05). The VAS results at the last follow-up were 0.67 ± 1.16, 2.45 ± 2.56 and 2.00 ± 2.69 for the lesion size < 15%, 15–30% and > 30%, respectively, and there were no significant differences (*P* > 0.05) too.

There were no significant differences in HHS results at the last follow-up between C1 group (82.71 ± 10.75) and C2 group (87.81 ± 10.02, *P* > 0.05). The HHS results at the last follow-up were 82.81 ± 11.77, 89.18 ± 7.73 and 92.00 ± 6.68 for the collapse degree ≤ 2 mm, 2–4 mm and > 4 mm, respectively, and there were no significant differences (*P* > 0.05). The HHS results at the last follow-up were 89.00 ± 10.15, 85.68 ± 11.32 and 88.67 ± 7.75 for the lesion size < 15%, 15–30% and > 30%, respectively, and there were no significant differences (*P* > 0.05) too.

There were no significant differences in WOMAC results at the last follow-up between C1 group (18.29 ± 12.93) and C2 group (11.00 ± 13.14, *P* > 0.05). The WOMAC results at the last follow-up were 19.19 ± 15.83, 7.91 ± 6.73 and 4.43 ± 5.35 for the collapse degree ≤ 2 mm, 2–4 mm and > 4 mm, respectively, and there was only a significant difference between the results for collapses ≤ 2 mm and > 4 mm (*P* < 0.05), and none of the others (*P* > 0.05). The WOMAC results at the last follow-up were 10.67 ± 11.59, 13.18 ± 14.29 and 11.44 ± 12.24 for the lesion size < 15%, 15–30% and > 30%, respectively, and there were no significant differences (*P* > 0.05).

There were no significant differences in iHOT-12 results at the last follow-up between C1 group (98.14 ± 14.31) and C2 group(101.89 ± 12.90, *P* > 0.05). The iHOT-12 results at the last follow-up were 95.81 ± 14.77, 106.45 ± 10.41 and 104.71 ± 8.30 for the collapse degree ≤ 2 mm, 2–4 mm and > 4 mm, respectively, and there were no significant differences (*P* > 0.05). The iHOT-12 results at the last follow-up were 109.33 ± 12.50, 99.41 ± 13.71 and 102.44 ± 11.61 for the lesion size < 15%, 15–30% and > 30%, respectively, and there were no significant differences (*P* > 0.05) too.

As for the Tonnis grade distribution at the last follow-up, group C1 has 1 at Grade 0, 3 at Grade 2, 3 at Grade 3, while group C2 has 1 at Grade 0, 6 at grade 1, 12 at Grade 2 and 8 at Grade 3, and the distribution difference was not statistically significant (*P* > 0.05). The collapse ≤ 2 mm group has 2 at Grade 0, 2 at Grade 1, 5 at Grade 2, 7 at Grade 3, while the group of 2-4 mm has 2 at Grade 1, 7 at Grade 2, 2 at Grade 3 and the group of > 4 mm has 2 at Grade 1, 3 at Grade 2 and 2 at Grade 3. As for the lesion size, group < 15% has 1 at Grade 0 and 2 at Grade 3, group 15–30% has 5 at Grade 1, 11 at Grade 2, 6 at Grade 3, while group > 30% has 1 at Grade 0, 1 at Grade 1, 4 at Grade 2 and 3 at Grade 3. There were no significant differences among them (*P* > 0.05).

**Fig. 4:**
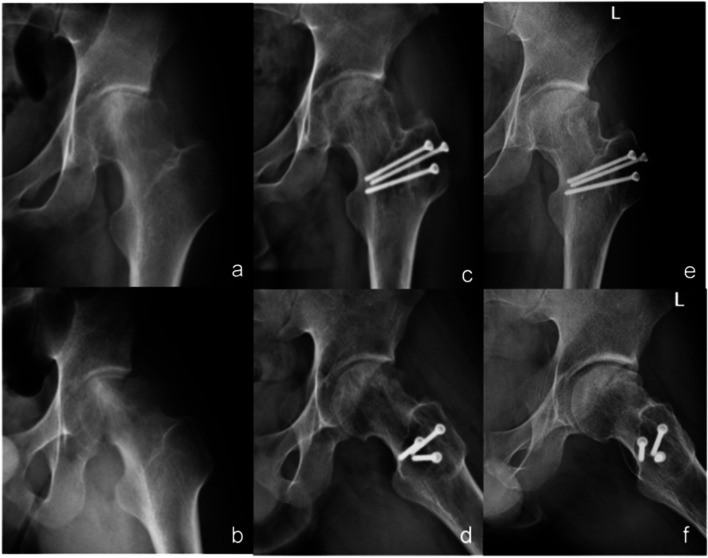
Radiographs of a 20-year-old male with idiopathic ONFH, ARCO IIIB, JIC C2. **a** and **b** A large area of bone density change can be seen on the X-rays (anterio-posterior and frog position) before the operation, and the femoral head is obviously collapsed and deformed. **c** and **d** X-rays (antero-posterior and frog position) of the patient with SHD combined with bone graft at 2 years after operation, and it can be observed that the collapse of the femoral head has not become more serious, and the bone density in the necrotic area has increased. **e** and **f** X-rays (antero-posterior and frog position) of the patient at six years after operation, it can be observed that the shape of the femoral head is maintained well, the collapse of the femoral head has not progressed, the density is increased, most of the necrotic area is repaired, the joint space remains normal, and there is no subluxation of the hip joint

A typical case is shown in Fig. 4.

## Discussion

Preserving the patient's own hip joint and prolonging the service life of the joint has always been the goal pursued by clinicians. In the West, the most commonly used methods for adult hip preservation are core decompression (CD) and bone grafting [31], but the collapse rate of such minimally invasive hip preservation surgery is as high as 50% at two years after operation [32]. In addition, for patients with large necrosis or ARCO stage III/IV, nonsurgical hip preservation or CD often cannot prevent progressive collapse of the femoral head [33–35]. THA is considered to be the ultimate method for advanced ONFH and secondary end-stage osteoarthritis [31]. For children with avascular necrosis of the femoral head, studies have shown that conservative treatment can achieve good results in a large proportion of patients [36–40]. However, for adolescents and young adults, there is no consensus on the best treatment for ONFH, and a large number of research reports are lacking. Although the survival time of THA is up to 25 years, for AYA patients, preserving their femoral head, delaying the development of the disease, and delaying the time of artificial joint replacement are still the most expected results. Studies have shown that in young patients, delaying the time of THA surgery has a positive significance for prolonging the use time of the prosthesis and improving clinical outcomes [41].

The SHD approach has been recognized by the majority of surgeons for its safety and its ability to fully expose the femoral head [42, 43]. At the same time, Ko believes that bone grafting can delay the natural progression of osteonecrosis, especially for teenagers [44]. Perhaps due to the rapid growth and development stage, the bone repair ability of children with ONFH is stronger. However, if you do not actively intervene after necrosis, AYA patients who are also capable of growth and development will quickly experience collapse and even disability, and then they have no choice but to choose THA [45–48]. Previous studies have shown that [49], compared with elderly patients, patients with an age of less than 30 years, necrosis in stages I-III, and smaller lesions can obtain better hip preservation effects. Therefore, when the patient is in the period of rapid bone repair and reconstruction, that is, the AYA population, whether the severity of necrosis will affect the efficacy of hip preservation is the focus of this study.

Contrary to the results reported in the literature, the results of this study showed that in the AYA population, the severity of preoperative osteonecrosis did not affect the clinical outcome of SHD combined with bone grafts. Even if the femoral head collapse of the patient is greater than or equal to 2 mm and the lesion size is relatively large, good short-term efficacy can still be achieved through this hip preservation surgery and this is consistent with our hypothesis. In this study, the lesion size and collapse degree were significantly reduced compared with those before surgery. The clinical scores of various functions and qualities of life were significantly improved, indicating that SHD combined with bone grafts can effectively treat AYA patients with ONFH. Regardless of the classifications and stages, this surgery can achieve good short-term clinical efficacy, delay the progression and increase the service life of the hip joints.

The results of subgroup analysis also confirmed it. In the results of subgroup analysis, both patients in JIC C1 and C2 groups could obtain better clinical function scores, and there was no difference in final osteoarthritis distribution. The final clinical function score and the distribution of osteoarthritis in patients with different lesion size can also obtain appreciable and similar results. Only patients with collapse ≤ 2 mm and > 4 mm differed in final WOMAC results. It is possible that the patients with severe collapse have continuously increased their acceptance of pain during the course of the disease, so that the effect of postoperative symptom improvement has been artificially amplified by the patient. This further supports our hypothesis that patients with severe collapse and extensive lesions are more deserving taking aggressive treatment to delay or even avoid THA.

We found that this may be related to the strong ability of bone repair and remodelling in AYA patients. After removing the necrotic bone, we implanted healthy autologous iliac bone and fixed it tightly so that the blood vessels could smoothly pass through from the normal bone into the iliac bone and finally reach the subchondral bone area to better promote bone repair and remodelling [50, 51]. This may also be the reason why there is no correlation between postiHOT-12 and preoperative lesion size and collapse degree. When the sample size is further expanded and the follow-up time is further extended, the results may change.

It's worth noting that the clinical scores of patients were significantly improved after surgery, and the trend of changes in the score results gradually slowed over time. It may suggest that we need to pay attention to the early postoperative functional recovery of patients, and temporary pain or limited range of motion may have long-term effects on the clinical function of patients. We recommend not rushing to walk with full weight on the ground, and it is best to perform rehabilitation training under the guidance of a doctor.

In this study, three patients were significantly worse and converted to THA nearly 2 years after surgery. All of them were JIC C2 preoperatively, with large lesion size and collapse over 2 mm. Obvious pain before surgery indicates severe fracture of the trabecular bone in the femoral head. In the early postoperative period, they did not strictly follow the doctor's instructions to perform protective weight-bearing walking, so the collapse of the femoral head progressed rapidly in the short term and was eventually irreversible. We think that there is a possibility of rapid progression of the disease within 2 years after surgery. After 2 years, the repair of necrosis within the head tends to be stable, and the impact on clinical symptoms and limb function will gradually decrease.

There are some limitations in this study. First, the sample size was small, which may have led to the failure of some relevant factors to test the impact on postiHOT-12 in the regression analysis. Second, the follow-up time was short, and the follow-up results within 1 year after surgery were unavailable, so we could only perform a rough analysis of the early postoperative trend of the disease. Finally, the results will be more convincing if a control group such as other surgical methods or populations can be matched. We will continue to observe its long-term efficacy and conduct further research.

## Conclusion

For AYA patients with ONFH, SHD combined with bone grafting may be an option for good short-term clinical outcomes. Moreover, preoperative HHS, WOMAC, iHOT-12, collapse degree, lesion size and JIC classification did not affect short-term clinical function and osteoarthritis. Young patients have stronger bone plasticity and better repair ability. Despite the obvious collapse and large lesion size, good effects can also be achieved by this surgery. Therefore, we suggest expanding the indications for hip preservation by SHD combined with bone grafts in AYA patients, regardless of the lesion size or the stage of the disease.

## Supplementary Information

**Additional file 1.** 

**Additional file 2.** 

## Data Availability

The datasets generated and/or analysed during the current study are not publicly available, because they are stored in the hospital office system, but are available from the corresponding author on reasonable request.

## Abbreviations

ONFH: Osteonecrosis of the Femoral Head
THA: Total Hip Arthroplasty
AYAs: Adolescents and Younger Adults
SHD: Surgical Hip Dislocation
VAS: Visual Analog Scale
HHS: Hip Harris Score
WOMAC: Western Ontario and McMaster Universities Osteoarthritis Index
ARCO: Association Research Circulation Osseous
JIC: Japanese Investigation Committee
CD: Core Decompression
BMI: Body Mass Index

## References

[1] Bernhard ME, Barnes CL, DeFeo BM, Kaste SC, Wang X, Lu Z (2021). Total Hip Arthroplasty in Adolescents and Young Adults for Management of Advanced Corticosteroid-Induced Osteonecrosis Secondary to Treatment for Hematologic Malignancies. J Arthroplasty.

[2] Karimova EJ, Rai SN, Howard SC, Neel M, Britton L, Pui CH (2007). Femoral head osteonecrosis in pediatric and young adult patients with leukemia or lymphoma. J Clin Oncol.

[3] Kawedia JD, Kaste SC, Pei D, Panetta JC, Cai X, Cheng C (2011). Pharmacokinetic, pharmacodynamic, and pharmacogenetic determinants of osteonecrosis in children with acute lymphoblastic leukemia. Blood.

[4] Mattano LA, Sather HN, Trigg ME, Nachman JB (2000). Osteonecrosis as a complication of treating acute lymphoblastic leukemia in children: a report from the Children's Cancer Group. J Clin Oncol.

[5] Ojala AE, Paakko E, Lanning FP, Lanning M (1999). Osteonecrosis during the treatment of childhood acute lymphoblastic leukemia: a prospective MRI study. Med Pediatr Oncol.

[6] Pui CH, Campana D, Pei D, Bowman WP, Sandlund JT, Kaste SC (2009). Treating childhood acute lymphoblastic leukemia without cranial irradiation. N Engl J Med.

[7] Sala A, Mattano LA, Barr RD (2007). Osteonecrosis in children and adolescents with cancer - an adverse effect of systemic therapy. Eur J Cancer.

[8] Salem KH, Brockert AK, Mertens R, Drescher W (2013). Avascular necrosis after chemotherapy for haematological malignancy in childhood. Bone Joint J.

[9] te Winkel ML, Pieters R, Hop WC, de Groot-Kruseman HA, Lequin MH, van der Sluis IM (2011). Prospective study on incidence, risk factors, and long-term outcome of osteonecrosis in pediatric acute lymphoblastic leukemia. J Clin Oncol.

[10] Mont MA, Jones LC, Hungerford DS (2006). Nontraumatic osteonecrosis of the femoral head: ten years later. J Bone Joint Surg Am.

[11] Evans JT, Evans JP, Walker RW, Blom AW, Whitehouse MR, Sayers A (2019). How long does a hip replacement last? A systematic review and meta-analysis of case series and national registry reports with more than 15 years of follow-up. Lancet.

[12] Ganz R, Gill TJ, Gautier E, Ganz K, Krugel N, Berlemann U (2001). Surgical dislocation of the adult hip a technique with full access to the femoral head and acetabulum without the risk of avascular necrosis. J Bone Joint Surg Br.

[13] Giordano V, Giordano M, Gloria RC, de Souza FS, di Tullio P, Lages MM (2019). General principles for treatment of femoral head fractures. J Clin Orthop Trauma.

[14] De Mauro D, Rovere G, Smakaj A (2021). Gibson approach and surgical hip dislocation according to Ganz in the treatment of femoral head fractures. BMC Musculoskelet Disord.

[15] Yao C, Yi N, Shen J, Du B, Sun G, Shu H (2017). Clinical reports of surgical dislocation of the hip with sequestrum clearance and impacting bone graft for grade IIIA-IIIB aseptic necrosis of femoral head (ANFH) patients. Oncotarget.

[16] Sun H, Wei B (2019). [Impacting bone graft via surgical hip dislocation approach versus core decompression and bone graft for avascular necrosis of femoral head at ARCO stage]. Zhongguo Xiu Fu Chong Jian Wai Ke Za Zhi.

[17] Wei Q, Pang F, Chen X, Yang P, He M, Fang B (2020). Clinical outcome of surgical hip dislocation combined with impacting bone graft and nonvascularized iliac flap implanting in the treatment of ARCO III osteonecrosis of the femoral head. Chin J Injury Repair and Wound Healing ( Electronic Edition).

[18] Xia T, Wei W, Liu J, Zhang C, Shen J (2019). Effectiveness comparison between impacting bone graft and rotational osteotomy via surgical hip dislocation approach for avascular necrosis of femoral head at ARCO stage. Zhongguo Xiu Fu Chong Jian Wai Ke Za Zhi.

[19] Zhuang Z, Wu Z, Xie Q, Gong Z, Lin X, Zhang H (2017). [Treatment of 22 Cases of Osteonecrosis of Femoral Head in Middle-aged and Young Adults with ARCO Stage III by Traditional Chinese Medicine Combined with Surgical Hip Dislocation and Bone Grafting]. Chinese J Trad Med Traum & Orthop.

[20] Seyler TM, Marker DR, Ulrich SD, Fatscher T, Mont MA (2008). Nonvascularized bone grafting defers joint arthroplasty in hip osteonecrosis. Clin Orthop Relat Res.

[21] Ricciardi BF, Sink EL (2014). Surgical Hip Dislocation: Techniques for Success. J Pediatr Orthop.

[22] Yoon B-H, Mont MA, Koo K-H (2020). The 2019 Revised Version of Association Research Circulation Osseous Staging System of Osteonecrosis of the Femoral Head. J Arthroplasty.

[23] Sheng D, Song Q, Zhang Q, He W, Chen DTL (2021). [Treatment of osteonecrosis of the femoral head at ARCO stage II and III of the young and middle-aged with autogenous iliac bone flap compression and bone grafting via surgical dislocation of the hip]. Chin J Bone Joint Injury.

[24] Liang D, Zhang L, Pei JXC (2021). [Comparison of clinical effectiveness of surgical hip dislocation vs. Orthopadische Chirurgie München approach with suppression of bone grafting in the treatment of pre - collapse osteonecrosis of the femoral head]. Orthopaedics.

[25] Tang Y, Li H, Yang Y, Li W, Xi J, Yue C (2020). Treatment of femoral head necrosis with surgical dislocation through fovea bone grafting combined with autologous bone marrow concentrate and platelet-rich plasma gel. Chin J Anat Clin.

[26] Steppacher SD, Sedlmayer R, Tannast M, Schmaranzer F, Siebenrock KA (2020). Surgical hip dislocation with femoral osteotomy and bone grafting prevents head collapse in hips with advanced necrosis. Hip Int.

[27] Zheng P, Li W, Weng S, Zhan Y, Chen L, Wu L (2020). Observation of the short- and medium-term effect of surgical hip dislocatio combined with surgery in the treatment of osteonecrosis of femoral head. Chin J Bone Joint Injury.

[28] Du M, Liu X, Qin G, He KPZ (2019). [Clinical efficacy of impaction bone-grafting after surgical hip dislocation and head and neck fenestration combined with Huoxue Bushen formula for middle-or late-stage osteonecrosis of the femoral head with kidney deficiency and blood stasis]. Guangxi Med J.

[29] Deng X, Liu J, Chen G, Yin Y, Yang S, Yue Y (2018). Surgical Hip Dislocation Approach for Treatment of Precollapse Stage of Femoral Head Necrosis. J Practical Orthopaedics.

[30] Liu S, Wei B, Guo XLJ (2018). [Platelet-rich plasma combined with surgical hip joint dislocation: A effective option for femoral head necrosis in the peri-collapse stage]. J Med Postgra.

[31] Kuroda Y, Okuzu Y, Kawai T, Goto K, Matsuda S (2021). Difference in Therapeutic Strategies for Joint-Preserving Surgery for Non-Traumatic Osteonecrosis of the Femoral Head between the United States and Japan: A Review of the Literature. Orthop Surg.

[32] Chotivichit A, Korwutthikulrangsri E, Pornrattanamaneewong C, Achawakulthep C (2014). Core decompression with bone marrow injection for the treatment of femoral head osteonecrosis. J Med Assoc Thai.

[33] Bedard NA, Callaghan JJ, Liu SS, Greiner JJ, Klaassen AL, Johnston RC (2013). Cementless THA for the treatment of osteonecrosis at 10-year follow-up: have we improved compared to cemented THA?. J Arthroplasty.

[34] Kim YH, Oh SH, Kim JS, Koo KH (2003). Contemporary total hip arthroplasty with and without cement in patients with osteonecrosis of the femoral head. J Bone Joint Surg Am.

[35] Mont MA, Cherian JJ, Sierra RJ, Jones LC, Lieberman JR (2015). Nontraumatic Osteonecrosis of the Femoral Head: Where Do We Stand Today? A Ten-Year Update. J Bone Joint Surg Am.

[36] Kerimoglu S, Citlak A, Baki C, Aydin H (2012). The long-term results of brace treatment in Perthes disease. Eklem Hastalik Cerrahisi.

[37] Killian JT, Niemann KM (1985). Preoperative skeletal traction in Legg-Perthes disease. South Med J.

[38] Aksoy MC, Cankus MC, Alanay A, Yazici M, Caglar O, Alpaslan AM (2005). Radiological outcome of proximal femoral varus osteotomy for the treatment of lateral pillar group-C Legg-Calve-Perthes disease. J Pediatr Orthop B.

[39] Thompson GH (2011). Salter osteotomy in Legg-Calve-Perthes disease. J Pediatr Orthop.

[40] Kanatli U, Ayanoglu T, Ozer M, Ataoglu MB, Cetinkaya M (2019). Hip arthroscopy for Legg-Calve-Perthes disease in paediatric population. Acta Orthop Traumatol Turc.

[41] Sultan AA, Khlopas A, Surace P, Samuel LT, Faour M, Sodhi N (2019). The use of non-vascularized bone grafts to treat osteonecrosis of the femoral head: indications, techniques, and outcomes. Int Orthop.

[42] Masse A, Aprato A, Alluto C, Favuto M, Ganz R (2015). Surgical hip dislocation is a reliable approach for treatment of femoral head fractures. Clin Orthop Relat Res.

[43] Abdelnasser MK, Refai O, Farouk O (2022). Surgical hip dislocation in fixation of acetabular fractures: Extended indications and outcome. Injury.

[44] Ko JY, Meyers MH, Wenger DR (1995). "Trapdoor" procedure for osteonecrosis with segmental collapse of the femoral head in teenagers. J Pediatr Orthop.

[45] Bali K, Sudesh P, Patel S, Kumar V, Saini U, Dhillon MS (2011). Pediatric femoral neck fractures: our 10 years of experience. Clin Orthop Surg.

[46] Inan U, Kose N, Omeroglu H (2009). Pediatric femur neck fractures: a retrospective analysis of 39 hips. J Child Orthop.

[47] Canale ST (1990). Fractures of the hip in children and adolescents. Orthop Clin North Am.

[48] Dhar SA, Ali MF, Dar TA, Sultan A, Butt MF, Kawoosa AA (2009). Delayed fixation of the transcervical fracture of the neck of the femur in the pediatric population: results and complications. J Child Orthop.

[49] Allsopp BJ, Hunter-Smith DJ, Rozen WM (2016). Vascularized versus Nonvascularized Bone Grafts: What Is the Evidence?. Clin Orthop Relat Res.

[50] Li ZQ, Chen D, Chen ZQ, Wei QS, Fang B, Zhang QW (2018). Treatment of collapsed traumatic osteonecrosis of the femoral head in adolescents using non-vascularized bone grafting via the trapdoor procedure. Int J Clin Exp Med.

[51] Pierce TP, Elmallah RK, Jauregui JJ, Poola S, Mont MA, Delanois RE (2015). A current review of non-vascularized bone grafting in osteonecrosis of the femoral head. Curr Rev Musculoskelet Med.

