# Supplementary material for: Outcomes of surgical hip dislocation combined with bone graft for adolescents and younger adults with osteonecrosis of the femoral head: a case series and literature review
Source: BMC Musculoskelet Disord. 2022 May 26;23:499. doi: 10.1186/s12891-022-05456-w (PMC9134689; doi:10.1186/s12891-022-05456-w)
Supplement: Supplementary file 2 — Additional file 2. [file 12891_2022_5456_MOESM2_ESM.doc]

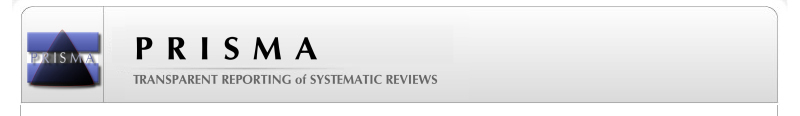
**PRISMA 2009 Flow Diagram**

**Screening**

**Included**

**Eligibility**

**Identification**

Records identified through database searching
(n = 1091 )

Pubmed (n=583) Embase (n=5)

Cochrane Library (n=9) CNKI(n=494)

Additional records identified through other sources
(n = 0 )

Total
(n = 1091 )

Screening on title and abstract

(n= 1085 )

Full-text articles assessed for eligibility
(n = 95 )

Articles excluded, with reasons
(n = 990 )

1. Animal/cadaver study(n=10)
2. Meta/Review(n=132)
3. Hip replacement/arthroplasty(n=101)
4. Guideline/Expert Experience(n=29)
5. Fracture(n=53)
6. Rehabilitation(n=8)
7. Perthes(n=29)
8. Hip dislocation(n=7)
9. Editor comment/Conference(n=37)
10. Nursing(n=15)
11. No full text(n=9)
12. Case report(n=8)
13. Thesis(n=130)
14. Other(n=422)

Articles included in review
(n = 13 )

Excluding duplicates
(n = 6 )
